# The microbiome of chronic rhinosinusitis: culture, molecular diagnostics and biofilm detection

**DOI:** 10.1186/1471-2334-13-210

**Published:** 2013-05-08

**Authors:** Sam Boase, Andrew Foreman, Edward Cleland, Lorwai Tan, Rachel Melton-Kreft, Harshita Pant, Fen Z Hu, Garth D Ehrlich, Peter-John Wormald

**Affiliations:** 1 Department of Surgery-Otorhinolaryngology, Head and Neck Surgery, University of Adelaide, Adelaide, Australia; 2 Center for Genomic Sciences, Allegheny-Singer Research Institute, Pittsburgh, PA, USA; 3 Department of Otorhinolaryngology, Head and Neck Surgery, The Queen Elizabeth Hospital, 28, Woodville Road, Woodville SA 5011, Australia

## Abstract

**Background:**

Bacteria and fungi are believed to influence mucosal inflammation in chronic rhinosinusitis (CRS). However their presence and relationship to disease is debated. This study used multiple detection methods to compare microbial diversity and microbial abundance in healthy and diseased sinonasal mucosa. The utility of contemporary detection methods is also examined.

**Methods:**

Sinonasal mucosa was analyzed from 38 CRS and 6 controls. Bacterial and fungal analysis was performed using conventional culture, molecular diagnostics (polymerase chain reaction coupled with electrospray ionization time-of-flight mass spectrometry) and fluorescence *in situ* hybridization*.*

**Results:**

Microbes were detected in all samples, including controls, and were often polymicrobial. 33 different bacterial species were detected in CRS, 5 in control patients, with frequent recovery of anaerobes. *Staphylococcus aureus* and *Propionibacterium acnes* were the most common organisms in CRS and controls, respectively. Using a model organism, FISH had a sensitivity of 78%, and a specificity of 93%. Many species were detected in both CRS and controls however, microbial abundance was associated with disease manifestation.

**Conclusions:**

This study highlights some cornerstones of microbial variations in healthy and diseased paranasal sinuses. Whilst the healthy sinus is clearly not sterile, it appears prevalence and abundance of organisms is critical in determining disease. Evidence from high-sensitivity techniques, limits the role of fungi in CRS to a small group of patients. Comparison with molecular analysis suggests that the detection threshold of FISH and culture is related to organism abundance and, furthermore, culture tends to select for rapidly growing organisms.

## Background

Chronic rhinosinusitis is a disease cluster with a significant societal burden, and despite extensive research efforts, has an unknown pathophysiology. There is emerging evidence that microorganisms play an important role in the exacerbation and perpetuation of mucosal inflammation. However, the microbial biodiversity in disease and controls is not well defined. Furthermore, the importance of microorganism abundance, and potential relationship to disease manifestation is unknown. To further our understanding of the role of microorganisms in CRS, it is important to comprehensively characterize the resident microbial community in healthy and diseased tissue and examine the specific host immunological responses to these organisms. Thus, we sought to characterize the microbial populations in CRS and controls to establish a basis for further species directed research into this heterogeneous disease. Only through such a systematic approach can we determine the importance, or otherwise, of these microorganisms in the disease phenotypes. Furthermore, comparative microbiome studies will provide important information for the selection of antimicrobial therapies, and enable the determination of the effectiveness of such treatments.

Increasingly, we are discovering that complex polymicrobial communities exist, especially at the host-environment interface such as mucosal surfaces, and the majority of these bacterial species are refractory to culture [1]. Many of these organisms are found to be residing in complex communities known as biofilms; communities of organisms surrounded by a self-produced exopolysaccharide matrix, irreversibly attached to a live or inert surface [2]. There have been recent advances in our understanding of biofilms in CRS, and their importance regarding disease evolution following treatment [3], increased postoperative infection [3], and altered host immune mechanisms [4]. Many biofilm organisms are resistant to culture [5], and their detection requires specialized techniques [6]. Phenotypic differences which occur between planktonic and biofilm based organisms, may contribute to the relative incapacity of biofilm associated organisms to grow on nutrient media [7,8].

Traditional culture-dependant techniques have been the mainstay of microbial diagnostics in CRS. However, the reliance of cultivation on nutrient media often results in ‘enrichment bias’ with detection of a narrow range of microbes which is not representative of the actual diversity present, particularly in environmental samples [9]. Competition between organisms during enrichment often results in dominance of one or two organisms with the fastest growth rates [9]. Selective media techniques use nutrient restriction to enhance or restrict growth of organisms based on inherent microbial characteristics for identification. In a complex microbial community such as the diseased sinonasal mucosa, the identification of every organism using this method would be exhaustive. Additionally, many organisms may not thrive on nutrient media once the advantages of biofilm structures, and symbiotic relationships are lost.

The objective of this study was to determine the relationship between disease manifestation, and the biodiversity and abundance of mucosal microorganisms in CRS patients and controls using broad-based molecular diagnostics and conventional culture. Additionally, we sought to determine the specificity of contemporary biofilm detection methods by comparing them with molecular detection techniques in the same patients. Whilst our understanding of the importance of these communities in CRS, and their capacity to benefit or harm the host is in it’s infancy, the investigation is fundamental to furthering our understanding of this disease.

## Methods

This prospective study was undertaken in the tertiary referral rhinology practice of the senior author (PJW), at the academic hospitals, Adelaide, South Australia. The study was approved by the Ethics of Human Research Committee, The Queen Elizabeth Hospital, South Australia, and 44 consecutive patients provided informed consented to involvement in the study. 38 patients met the definition of CRS as defined by the rhinosinusitis taskforce [10] having failed medical therapy necessitating the need for endoscopic sinus surgery (ESS). A control group consisted of 6 patients who had no clinical or radiological evidence of sinus disease. These patients were undergoing transnasal endoscopic procedures including trans-sphenoidal hypophysectomy for non-functioning adenomas (5 patients) or CSF leak repair (1 patient). Patients were excluded if less than 18 years of age, immunocompromised, or had decreased ciliary function such as cystic fibrosis and Kartagener’s syndrome. Other exclusion criteria included inadequate mucosa for analysis, no fungal or bacterial culture taken, and antibiotic or systemic corticosteroid used in the three weeks preceding surgery.

### Tissue collection

CRS patients had sinus mucosal tissue harvested from the ethmoid sinuses during ESS. Control patients had mucosa harvested from the posterior ethmoid and sphenoid as required to access relevant skull base pathology. Tissue was immediately stored in Dulbecco’s modified Eagle medium (Gibco, Invitrogen Corp., Grand Island, NY) without antibiotic or antimycotic, and transported on ice to the laboratory for further analysis. Mucus was harvested for histopathological examination, and for routine bacterial and fungal culture. In the absence of mucus, a middle meatal swab was taken for bacterial and fungal culture.

### Bacterial & fungal culture

Intraoperative swabs were transported to the laboratory (Oxoid Transport Swabs, Thermo-Fischer Scientific, Scoresby, Australia) and were streaked onto Columbia horse blood agar, and Chocolate agar (Thermo-Fisher Scientific). Fungal swabs were inoculated onto Sabouraud agar (Thermo-Fisher Scientific). Further nutrient restriction and testing was performed as required for identification.

### Biofilm analysis

Fluorescence *in-situ* hybridization (FISH) was performed on mucosa that had been stored at −80°C. Cryopreservation prior to FISH analysis of sinus mucosa has been validated in our department [11]. Defrosted samples were washed thoroughly in MilliQ water prior to hybridization to remove planktonic organisms. Two probes were utilized on separate pieces of mucosa - a *S. aureus* specific 16S sequence conjugated to Alexa-488 probe, and a pan-fungal 18S Alexa-488 probe. (AdvanDx, Woburn, MA). The manufacturer’s protocol was followed. Briefly, samples were fixed to glass slides, dehydrated in 90% ethanol, air dried, and hybridized at 55°C for 90 minutes. Samples were transported to Adelaide Microscopy for analysis using the Leica TCS SP5 Confocal Scanning Laser Microscope (Leica Microsystems, Wetzlar, Germany). An excitation of 488 nm with emission range of 495 – 540 nm was used to detect *S. aureus* and fungus. The entire sample was systematically scanned for biofilm elements. Axial Z stacks (0.5 μM) were recorded of representative areas to construct a three dimensional virtual images of the tissue, overlying mucus and biofilm.

### DNA extraction

An 8 × 8 mm square of mucosa was carefully dissected for each patient using sterile instruments under laminar flow conditions and stored at −80°C prior to DNA extraction. A 1 mm [3] piece of this tissue was placed into a sterile microcentrifuge tube containing 270 μL of ATL Lysis buffer (Qiagen, Germantown, MD, cat# 19076) and 30 μL proteinase K (Qiagen, cat# 19131). Samples were incubated at 56°C until lysis of the material was noted by visual inspection, then 100 μL of a mixture containing 50 μL each of 0.1 mm and 0.7 mm Zirconia beads (Biospec cat# 11079101z, 11079107zx respectively) were added to the samples which were then homogenized for 10 min at 25 Hz using a Qiagen Tissuelyser. Nucleic acid from the lysed sample was then extracted using the Qiagen DNeasy Tissue kit (Qiagen cat# 69506). 10 μL of each sample was loaded per well for both the Ibis Bacteria, Antibiotic Resistance, and Candida (BAC) and Fungal detection PCR plates (Abbott Molecular, cat# PN 05N13-01).

### Ibis T5000 analysis - PCR coupled with electrospray ionization mass spectrometry

The BAC detection plate contains 16 PCR primer pairs that collectively survey all bacterial organisms by using both omnipresent loci (eg. 16S rDNA sequences), as well as more taxa-specific targets (eg. the *Staphylococcus*-specific *tuf*B gene) as well as providing coverage for major antibiotic resistance genes and *Candida*. The fungal detection plate also uses 16 PCR primer pairs that collectively survey nearly all pathogenic fungal species. An internal calibrant consisting of synthetic nucleic acid template is also included in each well for both assays which controls for false negatives (eg. from PCR inhibitors) and enables a semi-quantitative analysis of the amount of template DNA present. PCR amplification was carried out as per Jiang and Hofstadtler [12]. The PCR products were then desalted in a 96-well plate format and sequentially electrosprayed into a time-of-flight mass spectrometer. The spectral signals were processed to determine the masses of each of the PCR products present with sufficient accuracy that the base composition of each amplicon could be unambiguously deduced [13]. Using combined base compositions from multiple PCRs, the identities of the pathogens and a semi-quantitative determination of their relative concentrations in the starting sample were established by using a proprietary algorithm to interface with the Ibis database of known organisms.

### Statistical analysis

Demographic data and species data where appropriate were reported as the mean +/− interquartile range. The Kruskal-Wallis test was used to compare multiple groups with Dunn’s post hoc test for non-parametric data. Sensitivity and specificity are presented with upper and lower 95% confidence intervals (CI). Genomes per sample are presented as mean (lower – upper 95% CI), and analysed using The Mann–Whitney *U* test. GraphPad Prism software (San Diego, CA) was used for statistical analysis, and a p-value of 0.05 was considered significant.

## Results

The majority of CRS patients had relatively severe disease on radiologic scoring, 66% had polyposis, and the majority were undergoing revision endoscopic sinus surgery, reflecting the tertiary nature of the surgical practice (see Table 1).

**Table 1:** Demographic & clinical data

	**CRS**	**Controls**
Number	38	6
Age*	41 (35–47)	44 (37–54)
Male/Female	22/16	2/4
Nasal polyposis (%)	25 (66%)	0
Lund-MacKay CT Score*	17 (15–20)	0
Revision Surgery (%)	25 (66%)	0
Smoking	2 (5%)	0
Aspirin Sensitivity	3 (8%)	0
Asthma	10 (26%)	1 (17%)

* Mean (Interquartile range).

### Number of species detected

Molecular organism detection using the Ibis T-5000 was positive in 100% of CRS patients and controls. A total of 33 different bacterial species were identified in CRS patients by the Ibis system, with a mean of 3.0 (2.0-4.0) species detected per patient (see Additional file 1: Table S1). In control patients, 5 different organisms were detected with a mean of 2.0 (1.0-3.0) per patient. There was a trend of increasing mean isolates per patient from controls 2.0 (1.0-3.0), CRS without nasal polyposis (CRSsNP) 2.5 (1.0-3.0), to CRS with nasal polyposis (CRSwNP) 3.2 (2.0-4.0) but this was not statistically significant (p > 0.05, Kruskal-Wallis test, Dunn’s post-hoc comparison). Ibis analysis detected fungi in only 4 CRSwNP patients, and no fungi were detected in CRSsNP patients or controls.

Conventional culture was positive in 73% of CRS patients, with an average of 1.3 organisms per patient detected. 12 different organisms were identified. Cultures were positive from 33% of control patients. Only one organism (*Staphylococcus epidermidis*) was cultured from this subject group.

### Diversity

30/38 (79%) CRS patients, and 3/6 (50%) of control patients, had more than one bacterial species detected on the mucosa using the Ibis molecular diagnostic. *Staphylococcus aureus* was the most commonly detected organism in CRS patients (23/38, 61%), followed by *Staphylococcus epidermidis* (21/38, 55%), and *Propionibacterium acnes* (14/38, 37%, see Table 2). *Nocardia asteroides* (9/38, 24%), *Haemophilus influenzae* (5/38, 13%) and *Pseudomonas aeruginosa* (3/38, 8%) were detected less commonly. In control patients, *Propionibacterium acnes* was the most commonly detected organism (5/6, 85%), followed by *Staphylococcus epidermidis*, present in 4/6 (67%) of patients. Less commonly detected were *Staphylococcus aureus* 2/6 (33%), *Nocardia asteroides* 1/6 (17%) and *Streptococcus agalactiae* 1/6 (17%). Anaerobic species were detected in 18/38 (47%) of CRS patients, and 5/6 (83%) of control patients.

**Table 2:** Molecular detection top ten organisms: detection frequency and prevalence

	**CRS**			**Control**		
	**N = 38**			**N = 6**		
	**No. detected (%)**	**Total genomes/sample**	**Mean genomes/sample**	**No. detected (%)**	**Total genomes/sample**	**Mean genomes/sample**
*Staphylococcus aureus*	23 (61%)	10549	459	2 (33%)	101	51
*Staphylococcus epidermidis*	21 (55%)	508	24	4 (67%)	21	5
*Propionibacterium acnes*	14 (37%)	2339	167	5 (83%)	621	124
*Nocardia asteroides*	9 (24%)	1587	176	1 (17%)	72	72
*Haemophilus influenzae*	5 (13%)	404	81	-	-	-
*Corynebacterium pseudodiphtheriticum*	4 (11%)	2121	530	-	-	-
*Streptococcus agalactiae*	4 (11%)	57	14	1 (17%)	3	3
*Moraxella catarrhalis*	3 (8%)	2931	977	-	-	-
*Pseudomonas aeruginosa*	3 (8%)	1852	617	-	-	-
*Streptococcus pneumoniae*	3 (8%)	1908	636	-	-	-
Mean microbial genomes per patient sample all detected organisms (lower - upper 95% CI)	870 (605–1136)			136 (−32 - 303)		

Conventional cultures produced polymicrobial results in 21/38 (55%) CRS patients, whereas no control patients had polymicrobial flora using this method. CRS patients yielded predominantly *S. aureus* (14/38) and *Staphylococcus epidermidis*/coagulase-negative *Staphylococcus* (CNS) (11/38). Only *Staphylococcus epidermidis*/CNS was cultivated from 2 control patients. No growth was reported for 9/38 CRS patients, and 4/6 control patients.

Fungi were rarely detected in CRSwNP patients regardless of technique used, and were not detected in CRSsNP patients or controls. Analysis with the Ibis biosensor showed only two species - *Aspergillus fumigatus* (3), and *Bipolaris papendorfii* (1). Culture detected only two of the Ibis fungal positive patients (both *Aspergillus fumigatus*) plus one *Penicillium chrysogenum*, and one *Trichosporon* in other patients, which were not detected by the biosensor. Fungal-specific FISH analysis was positive in the three *A. fumigatus* patients detected by the Ibis biosensor, the *P. chrysogenum* culture-positive patient, and two additional patients, which were negative by the other techniques. Regardless of detection technique, all patients in whom fungi was detected had nasal polyposis.

### Detection of *S. Aureus* using ibis biosensor, FISH, and culture

**Figure 1:**
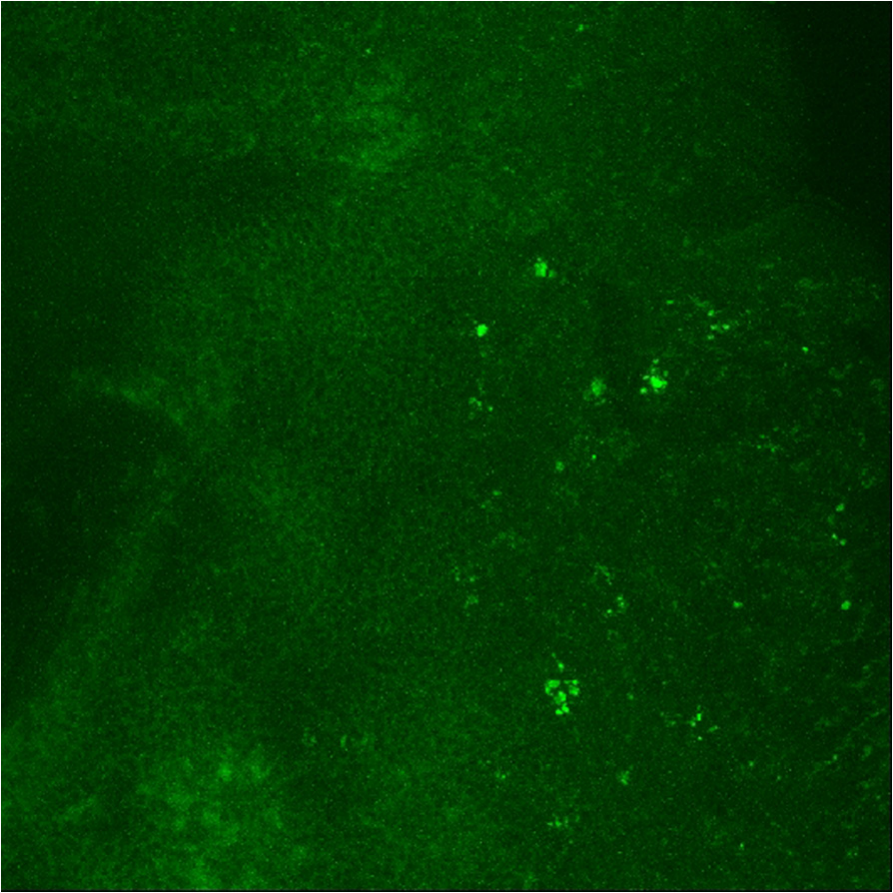
**Staphylococcus aureus FISH probe tagged with Alexa 488, analyzed with confocal scanning laser microscopy at 80× magnification.**

We used *S. aureus* as a model organism for studying the characteristics of the three different detection methods. The biosensor detected *S. aureus* in 61% of CRS patients, biofilm was positive in 50% (see Figure 1), and conventional culture was positive in 37%. FISH analysis had a sensitivity of 78% (±18) for the detection of *S. aureus* compared to Ibis, with a specificity of 93% (± 6). In contrast, conventional culture detected *S. aureus* with a sensitivity of 61% (±17), and a specificity of 93% (+16). *S. aureus* was detected in control patients only by molecular detection (2/6, 33%). Neither conventional culture nor FISH analysis was positive for *S. aureus* in these patients.

### Relative microbial density

We determined the relative numbers of the bacterial species using the Ibis biosensor, which provides a genomes/well measure. Results for the top ten organisms are shown in Table 2. The most commonly detected organisms in CRS patients, *S. aureus* and *S. epidermidis*, had markedly different microbial densities, with *S. aureus* organisms present in much higher numbers than *S. epidermidis* based on bacterial genomes detected. Some of the less commonly detected organisms such as *Corynebacterium pseudodiphtheriticum, Moraxella catarrhalis, Streptococcus pneumoniae,* and *Pseudomonas aeruginosa,* were found in relatively high numbers when detected on the mucosa. The abundance of *S. aureus* was much greater in CRS patients compared to controls, however other organisms such as *S. epidermidis* and *P. acnes* were detected in similar quantities in both patient groups. When all organisms were considered, CRS patients had significantly greater bacterial genomes per sample than control patients (p < 0.05, Mann–Whitney *U* test - see Table 2).

### Are FISH and culture detection related to microbial abundance

**Figure 2:**
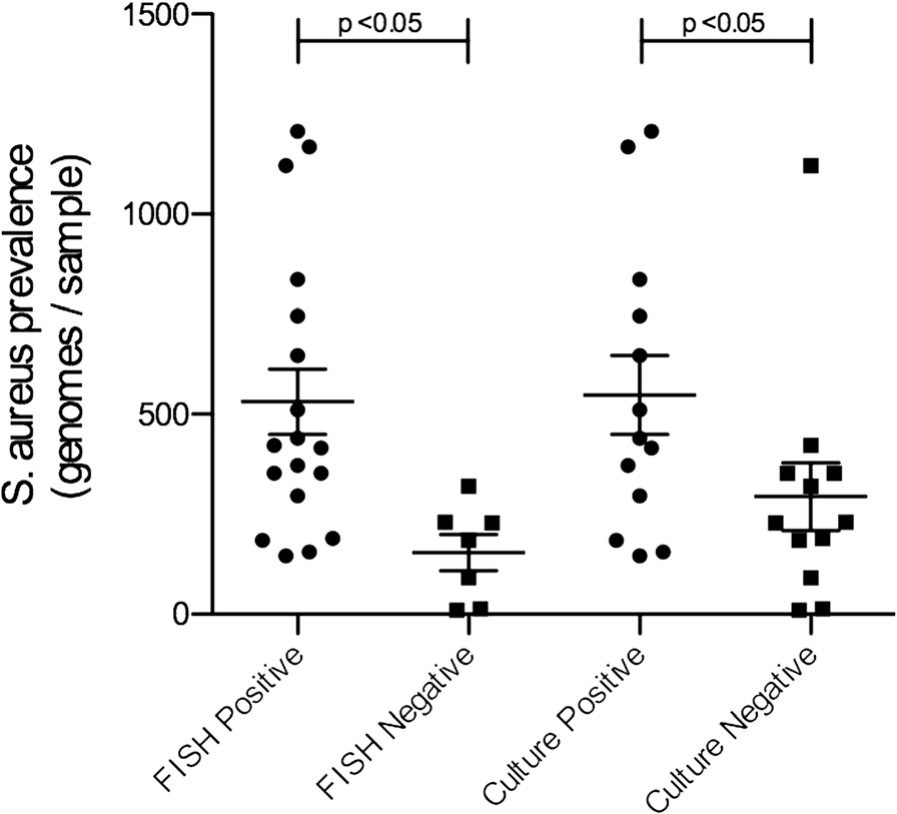
**The relationship between *S. aureus *abundance, and detection by FISH and culture.** The detection of *S. aureus* by FISH and culture is dependant on the abundance of the bacteria as measured by the number of bacterial genomes present in the sample.

We again used the organism *S. aureus* to compare detection techniques based on microbial numbers. The quantitative genomic analyses of the 25 (CRS and control) samples in which *S. aureus* was detected by the Ibis biosensor, were compared to the detection of the organism by FISH and conventional culture to determine if these latter detection methods are dependent on the abundance of organisms to provide a positive finding (see Figure 2). The number of *S. aureus* genomes per sample was significantly higher in those specimens that tested positive for *S. aureus* by FISH and conventional culture (p < 0.05, Kruskal-Wallis test, Dunn’s post-hoc comparison). However there was no statistically significant difference between FISH and culture detection sensitivity based on *S. aureus* genomes per sample (p > 0.05, see Figure 1).

## Discussion

This study compares the microbial bioburden in CRS patients, with healthy controls using three markedly different, but complementary detection techniques. We have shown that conventional laboratory culture has a tendency to polarize the detected microbes, selecting for abundant, rapidly growing aerobic organisms with favourable growth characteristics, such as *S. aureus* and *S. epidermidis*. Biosensor molecular detection allows a more comprehensive analysis of the microbial community and has the advantage of not requiring *a priori* knowledge of the flora. *S. aureus* was the most commonly detected organism in CRS patients, and was relatively more abundant in CRS patients compared to controls. It was detected with high sensitivity with FISH compared to the biosensor, but at lower levels by conventional culture. We have shown that molecular quantification can provide additional information with respect to microbial abundance, and comparisons between health and disease may assist our understanding of the role of these organisms in pathogenesis. Additionally we have shown that the detection of organisms using FISH and conventional culture is significantly dependant on microbial abundance as measured using molecular quantification. Fungi were present uncommonly, in a select group of nasal polyp patients.

*Staphylococcus aureus* was the most prevalent organism in CRS patients using molecular detection, followed by *Staphylococcus epidermidis* and *Propionibacterium acnes*. When the microbial density was compared for these three organisms between CRS and control patients, *S. aureus* was present at approximately 10 times higher genomes per sample in CRS patients, whereas *S. epidermidis* and *P. acnes* were found in similar abundance between both patient groups. The increased bioburden of *S. aureus* in the disease group is of particular interest, as it is emerging as a prominent disease modifying organism in CRS and it’s presence in patients has important clinical implications [14,15]. The capacity of *S. aureus* to exist within dense mucosal biofilms has been documented [11,16], which may explain the variable detection from clinical specimens using traditional culture techniques.

A multitude of other studies have recently reported CRS microbiological data using a variety of techniques, including molecular diagnostics and FISH. Comparison with these studies is of interest to further our understanding of this disease. In the current study, *Haemophilus influenzae* was detected at relatively low levels (13%) in CRS patients, and was not detected in controls. This is in agreement with Stephenson *et al.,* who detected *H. influenzae* in 17% of CRS patients [17], but contrasts with earlier FISH based studies which proposed it as the dominant organism in CRS [18,19]. *Pseudomonas aeruginosa* was also found infrequently in the current study (8%), confirming the findings of Stephenson *et al.* These results contrast with a recent molecular study which found *P. aeruginosa* to be the dominant organism in CRS patients, however control patients were not assessed [20]. The disparity in organism profiles between studies may reflect regional variation, patterns of antimicrobial use, methodological differences, or disease severity patterns.

The disease burden in the current study was relatively high as measured by the radiological severity and the rate of non-primary surgery, which were at least twice as high as others [17,20], reflecting the tertiary nature of the practice. Some microbial biofilms have been associated with a more severe disease process than others [16], and the microbial community in this study may reflect some bias towards the more severe end of the CRS spectrum. However, this is arguably the population which is most resistant to current treatment paradigms, and therefore of utmost importance to investigate.

The high prevalence of anaerobes detected in CRS patients is in agreement with previous molecular studies [17,20], however we also found high rates in controls, with similar abundance, casting doubt on a direct pathogenic role. There was poor agreement between the Ibis biosensor and conventional culture data for anaerobes, which reflects the paucity of culture-based studies reporting anaerobic species in CRS. These different detection methodologies seem to have comparable efficacy at detecting abundant, fast growing organisms. The pathologic importance of these species remains to be determined however.

Through the use of molecular detection we are beginning to understand the natural flora of non-diseased sinuses. Contrary to previous reports of sterility [21,22], we have shown that all healthy sinuses in this study are associated with a microbial community, and many of these organisms are also found in diseased sinuses. Nevertheless, we have shown that the abundance of organisms is significantly greater in CRS patients compared to controls - a phenomenon which requires further investigation with greater numbers of patients.

The cultivation of microorganisms using traditional culture has many pitfalls which may explain it’s limited utility in describing polymicrobial communities such as that within diseased sinuses. To be detected, organisms must grow on media after being removed from the native mucosal surface, with significant environmental changes in temperature, pH, nutrient sources, and without the complex dynamics of polymicrobial communities and host immune systems. Molecular methods such as the Ibis biosensor offer great potential for analysis of microbial diversity in CRS, which is untempered by the limitations of conventional culture. Molecular methods are sensitive and accurate and provide a more complete view of the bacterial communities present, and even provide a molecular antibiogram to guide treatment decisions. An important caveat however, is the capacity of molecular techniques to detect the genetic material of non-viable microorganisms. This must be taken into consideration when comparing between these, and culture-based techniques. Access to molecular detection instrumentation and expertise may also limit it’s utility in the clinical setting, but this is rapidly improving.

The formation of biofilms also impacts on the culture rates from patients. Organisms that form these immobilized consortia often undergo phenotypic transformations with reduced metabolic activity, which impacts the capacity to grow on selective media [2]. Using the model organism *S. aureus*, we have shown significantly increased detection rates using both FISH and molecular detection compared to cultivation techniques. Similar to previous studies [11,23,24], biofilm was not detected in the control patients, despite detection of *S. aureus* in 2/6 controls by the Ibis biosensor. *In situ* hybridisation techniques have previously been reported to have reduced sensitivity in detecting low copy number nucleic acids of scarce bacteria [1]. In an attempt to counter this, we have employed protein nucleic acid (PNA) FISH which has higher affinity and stability for microbial DNA sequences than conventional FISH probes [25]. Despite this however, some of the CRS patients, and both controls with *S. aureus* detected at low DNA copy number by Ibis, were not detected by FISH. This limitation not with standing, we have shown a good correlation between FISH and biosensor detection of *S. aureus* in CRS patients, with high sensitivity and specificity, suggesting it has good clinical and research applicability.

The prevalence of fungi in CRS and healthy control patients has long been debated [26]. Many studies have found a predominance of fungi in both patient groups when the nasal cavity is sampled [27-29]. It is possible that these studies are detecting inhaled environmental fungi, which is trapped within nasal mucus en-route to the oropharynx. When sinus mucosa is specifically analysed, studies suggest a lesser prevalence in CRS patients and absence in controls [30,31]. The characterization of fungal species in CRS is far less advanced than for bacteria, and whether or not certain fungal species are more prevalent or important in CRS is still unknown. Therefore we employed a pan-fungal FISH probe to detect all species, in conjunction with culture and molecular detection. We found fungi in a small proportion of the sinus mucosa of CRS patients using all three detection methods, and an absence in control patients. The three methods showed similar sensitivity. Fungi may be playing a role in this small subset of CRS patients, all of whom had nasal polyposis. This study refutes the theory that current culture methods are insensitive and missing a large proportion of patients in whom fungi are playing a central role.

The importance of understanding the complex polymicrobial communities in the sinuses is highlighted by the concept of dysbiosis, where organisms interact in positive (mutualistic) or negative ways to alter the local community, and interaction with the host. There is evidence to suggest that microbial diversity is important for health [32], and a reduced diversity with increased abundance is associated with chronic inflammation and poor healing [33]. There is also literature to suggest that host genetics and immunity strongly influence the composition of the mucosal microflora [34]. Microbial communities inhabiting mucosal surfaces such as the gastrointestinal tract can result in a significant mutualism including local immune homeostasis [35,36], and protection from pathogens through processes such as nutrient consumption, occupation of attachment sites, and secretion of antimicrobial substances [37]. *Propionibacterium acnes,* which was found in more than 80% of control patients in the current study, has been shown to produce bacteriocins which have antibacterial and antifungal activity which may be protective against pathogens [38]. Competition between microbes on mucosal surfaces can result in selection of virulence factors which can be detrimental to the host [39]. In an elegant model of polymicrobial interactions, Sibley *et al.*, have shown that avirulent organisms can enhance the pathogenicity of other organisms, highlighting the importance of comprehensive community analysis to investigate disease [40].

It is possible that disruption of mutualistic relationships, through shifts in mucosal-associated microbial composition, could contribute to the onset, progression, or recalcitrance of CRS. For example, the change in microbial dynamics during a viral upper respiratory tract infection or acute bacterial exacerbation of CRS. The often protracted use of antibiotics in our patients may also have detrimental sequelae on the microbial balance in CRS which requires further substantiation. There is poor understanding of such mechanisms in CRS, but they are an active focus of research at present. The first step in this journey is to greatly improve our knowledge of the mucosal microbial communities in CRS patients and controls.

Future directions for research should examine larger populations of CRS patients to characterize the microbiome of the different CRS phenotypes in comparison with healthy controls. Other molecular techniques, such as 16S rRNA gene pyrosequencing and quantitative real-time PCR will provide greater resolution for more detailed microbiome studies. Furthermore, longitudinal molecular studies evaluating the effect of antibiotics, endoscopic sinus surgery, and topical treatments on microbial diversity and abundance in CRS patients would be invaluable. Investigating the relationships within the microbial communities, and their interactions with the host immune system in health and disease, will ultimately lead to a greater understanding of the pathogenesis of chronic rhinosinusitis.

## Conclusion

This study has demonstrated some important hallmarks regarding the microbiome of chronic rhinosinusitis, and control sinuses. The healthy sinus is not sterile, and it appears that not only prevalence, but also abundance, of organisms is critical in determining the disease state. Comparison with molecular analysis suggests that the detection threshold of FISH and culture is related to organism abundance and, furthermore, culture tends to select for rapidly growing organisms.

## Competing interests

PJW receives royalties from Medtronic and is a consultant for Neilmed. GDE is a consultant for Stryker and Medtronic. SB, AF, EC, LWT, RM-K, HP & FZH have no competing interests. Funding: nil received.

## Authors’ contributions

SB - Project design, tissue collection & analysis, fungal and bacterial biofilm preparation & imaging, image analysis, statistical analysis, manuscript preparation. AF - Project design, assistance with biofilm analysis, manuscript editing. EC - Assistance with biofilm analysis, manuscript editing. LWT - Project co-supervision, manuscript editing. RM-K - Ibis sample preparation & analysis. HP - Project co-supervision, manuscript editing. FZH - Ibis analysis & interpretation. GDE - Ibis analysis & interpretation, manuscript editing. PJW - Principal project supervision, surgical procedures, manuscript editing. All authors read and approved the final manuscript.

## Pre-publication history

The pre-publication history for this paper can be accessed here:

http://www.biomedcentral.com/1471-2334/13/210/prepub

## Supplementary Material

Additional file 1: Table S1 Ibis Microorganism detection, CRSwNP = Chronic rhinosinusitis with nasal polyps, CRSsNP = Chronic rhinosinusitis without nasal polyps. Click here for file
