# Supplementary material for: The microbiome of chronic rhinosinusitis: culture, molecular diagnostics and biofilm detection
Source: BMC Infect Dis. 2013 May 8;13:210. doi: 10.1186/1471-2334-13-210 (PMC3654890; doi:10.1186/1471-2334-13-210)
Supplement: Additional file 1: Table S1 — Ibis Microorganism detection, CRSwNP = Chronic rhinosinusitis with nasal polyps, CRSsNP = Chronic rhinosinusitis without nasal polyps. [file 1471-2334-13-210-S1.docx]

| **Patient** | **Disease Classification** | **Ibis Bacterial Detection** | **Genomes per Sample** |
| --- | --- | --- | --- |
| 1 | CRSsNP | *Propionibacterium acnes*  *Moraxella catarrhalis*  *Staphylococcus epidermidis* | 72  1255  5 |
| 2 | CRSwNP | *Staphylococcus aureus*  *Escherichia coli*  *Pseudomonas aeruginosa*  *Streptococcus agalactiae* | 372  24  24  1 |
| 3 | CRSsNP | *Nocardia asteroides* | 30 |
| 4 | CRSwNP | *Actinobacillus pleuropneumoniae*  *Propionibacterium acnes*  *Anoxybacillus flavithermus*  *Staphylococcus epidermidis*  *Corynebacterium sp.*  *Lactobacillus acidophilus* | 1709  69  5  10  602  13 |
| 5 | CRSwNP | *Staphylococcus epidermidis*  *Propionibacterium granulosum* | 50  99 |
| 6 | CRSwNP | *Staphylococcus aureus*  *Haemophilus influenzae*  *Nocardia asteroides*  *Streptococcus agalactiae*  *Staphylococcus epidermidis*  *Streptococcus sanguinis* | 1208  104  37  5  5  3 |
| 7 | CRSwNP | *Staphylococcus aureus*  *Staphylococcus epidermidis*  *Haemophilus influenzae* | 746  57  4 |
| 8 | CRSsNP | *Staphylococcus aureus*  *Nocardia asteroides*  *Corynebacterium pseudodiphtheriticum*  *Lactobacillus salivarius* | 296  48  658  108 |
| 9 | CRSwNP | *Staphylococcus aureus* | 440 |
| 10 | CRSwNP | *Staphylococcus epidermidis*  *Propionibacterium acnes*  *Moraxella catarrhalis*  *Pediococcus pentosaceus* | 56  925  2537  12 |
| 11 | CRSsNP | *Prochlorococcus marinus*  *Propionibacterium acnes*  *Staphylococcus epidermidis*  *Corynebacterium pseudodiphtheriticum*  *Nocardia asteroides*  *Streptococcus agalactiae* | 424  62  50  327  133  50 |
| 12 | CRSsNP | *Staphylococcus aureus*  *Propionibacterium acnes*  *Staphylococcus epidermidis* | 185  232  3 |
| 13 | CRSwNP | *Staphylococcus aureus* | 1122 |
| 14 | CRSsNP | *Staphylococcus aureus*  *Fusobacterium necrophorum*  *Enterococcus faecium* | 231  199  10 |
| 15 | CRSsNP | *Staphylococcus aureus*  *Haemophilus sp.* | 511  280 |
| 16 | CRSsNP | *Staphylococcus aureus*  *Haemophilus influenzae*  *Streptococcus gordonii* | 230  24  6 |
| 17 | CRSsNP | *Staphylococcus epidermidis*  *Staphylococcus lugdunensis* | 30  1 |
| 18 | CRSwNP | *Staphylococcus aureus*  *Propionibacterium acnes* | 647  61 |
| 19 | CRSwNP | *Staphylococcus aureus*  *Streptococcus pyogenes*  *Francisella tularensis*  *Nocardia asteroids* | 415  353  1912  292 |
| 20 | CRSwNP | *Staphylococcus aureus*  *Propionibacterium acnes*  *Staphylococcus epidermidis*  *Nocardia asteroides*  *Staphylococcus lugdunensis* | 157  98  13  445  8 |
| 21 | CRSwNP | *Staphylococcus epidermidis*  *Staphylocccus aureus*  *Escherichia coli*  *Citrobacter koseri* | 50  320  1161  65 |
| 22 | CRSwNP | *Staphylococcus aureus*  *Propionibacterium acnes*  *Streptococcus pneumoniae*  *Staphylococcus epidermidis* | 1169  276  20  8 |
| 23 | CRSwNP | *Propionibacterium acnes*  *Staphylococcus epidermidis*  *Moraxella catarrhalis*  *Corynebacterium pseudodiphtheriticum* | 119  1  269  275 |
| 24 | CRSsNP | *Propionibacterium acnes* | 9 |
| 25 | CRSwNP | *Staphylococcus aureus*  *Staphylococcus epidermidis*  *Nocardia asteroides* | 353  6  123 |
| 26 | CRSwNP | *Propionibacterium acnes*  *Staphylococcus epidermidis*  *Corynebacterium pseudodiphtheriticum* | 49  20  259 |
| 27 | CRSsNP | *Staphylococcus aureus* | 836 |
| 28 | CRSwNP | *Staphylococcus epidermidis*  *Propionibacterium acnes*  *Slackia exigua* | 109  50  493 |
| 29 | CRSsNP | *Staphylococcus aureus* | 422 |
| 30 | CRSwNP | *Staphylococcus aureus*  *Haemophilus influenzae*  *Nocardia asteroides* | 147  16  19 |
| 31 | CRSwNP | *Streptococcus pneumoniae*  *Staphylococcus aureus*  *Staphylococcus epidermidis* | 1826  185  2 |
| 32 | CRSwNP | *Haemophilus influenzae*  *Staphylococcus epidermidis*  *Streptococcus pneumoniae*  *Nocardia asteroids*  *Actinobacillus pleuropneumoniae* | 256  16  62  466  3 |
| 33 | CRSwNP | *Pseudomonas aeruginosa*  *Staphylococcus epidermidis* | 1729  15 |
| 34 | CRSwNP | *Pseudomonas aeruginosa* | 99 |
| 35 | CRSsNP | *Propionibacterium acnes*  *Staphylococcus aureus*  *Staphylococcus epidermidis* | 312  14  1 |
| 36 | CRSwNP | *Staphylococcus aureus*  *Staphylococcus epidermidis*  *Streptococcus agalactiae* | 353  1  1 |
| 37 | CRSwNP | *Lactobacillus sp.* | 806 |
| 38 | CRSwNP | *Staphylococcus aureus* | 190 |
| 39 | Control | *Propionibacterium acnes*  *Staphylococcus epidermidis*  *Streptococcus agalactiae* | 445  6  3 |
| 40 | Control | *Nocardia asteroides*  *Propionibacterium acnes*  *Staphylococcus epidermidis* | 72  11  5 |
| 41 | Control | *Propionibacterium acnes* | 40 |
| 42 | Control | *Staphylococcus epidermidis*  *Propionibacterium acnes*  *Staphylococcus aureus* | 5  101  10 |
| 43 | Control | *Propionibacterium acnes* | 24 |
| 44 | Control | *Staphylococcus aureus* | 91 |

**Additional file 1: Table S1:** Ibis Microorganism detection, CRSwNP = Chronic rhinosinusitis with nasal polyps, CRSsNP = Chronic rhinosinusitis without nasal polyps,
